# Examining the Pre- and Post-percutaneous Coronary Intervention Blood Pressure Variability Using Ambulatory Blood Pressure Monitoring in Patients With Stable and Unstable Coronary Artery Disease

**DOI:** 10.7759/cureus.60465

**Published:** 2024-05-16

**Authors:** Abhisekh Chetal, Gurveer Singh Raien, Akhilesh Bandhu Gupta, Ajay Mishra, Neymat Kaur, Isha Rani, Anmol Goyal

**Affiliations:** 1 Department of General Medicine, Maharishi Markandeshwar College of Medical Science and Research, Ambala, IND; 2 Department of General Medicine, Eras Lucknow Medical College and Hospital, Lucknow, IND; 3 Department of Community Medicine, Adesh Medical College and Hospital, Shahbad, IND; 4 Department of Biochemistry, Maharishi Markandeshwar College of Medical Science and Research, Ambala, IND; 5 Department of Community Medicine, Maharishi Markandeshwar College of Medical Science and Research, Ambala, IND

**Keywords:** ambulatory blood pressure monitoring (abpm), percutaneous coronary intervention (pci), myocardial infarction (mi), nighttime, daytime, coronary artery disease (cad), unstable cad, stable cad, mi, dipping pci

## Abstract

Introduction

The World Health Organization has drawn attention to the fact that coronary artery disease (CAD) is our modern "epidemic." Nowadays, sudden death during sleep has become prevalent due to a lack of oxygen supply to the heart. CAD causes more deaths and disabilities and incurs greater economic costs than any other illness in the developed world. The prevalence of cardiovascular disorders and heart disease is on the rise in India. Hypertension is one of the leading risk factors for all cardiovascular diseases. This study aims to compare blood pressure variability before and after percutaneous coronary intervention (PCI), using ambulatory blood pressure monitoring (ABPM) in patients with stable and unstable CAD.

Materials and methods

This prospective observational study was conducted among 52 patients with stable and unstable CAD, admitted to the medicine department, who required PCI at a tertiary care hospital. Before and after PCI, the same antihypertensive drugs were orally administered. ABPM was performed before PCI and one day after PCI. ABPM was conducted every 30 minutes during the day and every 60 minutes during the night over a 24-hour period using a mobil-o-graph (IEM, Germany). The results of the observed parameters were analyzed using the HMS Client-Server 4.0 system (Informer Technologies, Inc., Los Angeles, USA). The collected data were analyzed using SPSS Statistics version 21.0 software (IBM Corp. Released 2012. IBM SPSS Statistics for Windows, Version 21.0. Armonk, NY: IBM Corp.).

Results

Out of 52 patients, 28 (53.8%) had stable CAD and 24 (46.2%) had unstable CAD. The mean age of patients with stable and unstable CAD was 56.64±9.44 and 57.04±12.36 years, respectively. The majority of patients with stable (67.9%) and unstable CAD (62.5%) were males. Various other variables were considered, such as lipid profile, blood sugar, cardiac troponin-I, and medical history, including hypertension and type 2 diabetes mellitus. Among stable CAD patients, a comparison between pre- and post-PCI systolic blood pressure (SBP) did not show a significant difference in all SBP measurements (p>0.05). However, the mean diurnal index was significantly lower following PCI compared to before PCI (p=0.019). Among unstable CAD patients, a comparison between pre- and post-PCI SBP showed a significant change in peak daytime, average daytime, and diurnal index (p<0.05). For all other SBP measurements, the difference between pre- and post-PCI measurements was not statistically significant (p>0.05). In patients with stable CAD, a statistically significant change in diastolic blood pressure (DBP) following PCI was observed for peak daytime, peak nighttime, and average nighttime values. In contrast, for patients with unstable CAD, a statistically significant change in DBP following PCI was observed for peak daytime, peak nighttime, and minimum daytime values (p<0.05). Statistically, post-PCI, there was no significant difference between the two groups for SBP and DBP measurements (p>0.05). Additionally, there was no significant difference between the two groups pre- and post-PCI in the pattern of dipping.

Conclusion

A comparison of the ABPM before and after PCI showed that, within 48 hours post-PCI, the ambulatory blood pressure indicators did not differ statistically from those before PCI.

## Introduction

Coronary artery disease (CAD) is a pathological process characterized by atherosclerotic plaque accumulation or transient vasoconstriction in one or more of the epicardial coronary arteries, leading to a mismatch between myocardial oxygen demand and supply [1]. Stable CAD refers to a reversible supply/demand mismatch related to ischemia, a history of myocardial infarction (MI), or the presence of plaque documented by catheterization or computed tomography angiography. Patients are considered stable if they are asymptomatic or if their symptoms are controlled by medications or revascularization [2,3]. Unstable CAD, on the other hand, involves an imbalance of oxygen supply and demand available to the myocardium. This imbalance sometimes causes symptoms such as new-onset exertional angina, pre-existing angina that is refractory to nitroglycerin, or angina at rest. Unstable CAD is thus characterized by an acute onset of angina and is one of the recognized components of acute coronary syndrome (ACS), along with non-ST-elevation MI [4].

CAD is the most common, serious, chronic, and life-threatening illness in the United States. Thirteen million people have CAD, over six million have angina pectoris, and more than seven million have sustained MI. A high-fat and energy-rich diet, smoking, and a sedentary lifestyle are associated with the emergence of CAD. In the United States and Western Europe, it is growing among low-income groups rather than high-income groups (who are adopting more healthy lifestyles), while primary prevention has delayed the disease until later in life in all socioeconomic groups [5]. The 2021 Heart Disease and Stroke Statistics update of the American Heart Association has recently reported that 26.1 million persons aged ≥20 years in the USA, comprising 9.3% of the total population in that age range, have coronary heart disease [6].

India bears almost 60% of the world's heart disease burden. The average age of patients with heart disease in India is at least five to eight years lower (60 years) compared to developed countries (63-68 years), and it is slipping further into the mid-50s. Moreover, Indians are also more likely to have types of heart disease that lead to worse outcomes, such as ischemic heart disease, a condition characterized by a reduced blood supply to the heart [7].

Considering blood pressure variability as an indicator of cardiovascular disease risk, percutaneous coronary intervention (PCI) was performed on both stable and unstable CAD patients. It is important to understand whether PCI is helpful in monitoring blood pressure variability patterns in CAD patients and, in turn, the associated risk of adverse cardiovascular events. Although blood pressure variability in CAD patients has been assessed in various contexts, the distinction between stable and unstable CAD and the effects of percutaneous intervention on these patients have not been extensively studied. Therefore, the present study was conducted to compare blood pressure variability and the dynamic characteristics and trends of ambulatory blood pressure monitoring (ABPM) in patients with stable and unstable CAD undergoing treatment before and after PCI. Additionally, this study aims to assess blood pressure variability with ABPM within 48 hours of admission in CAD patients before and after PCI.

## Materials and methods

The prospective observational study was conducted on patients with stable and unstable CAD requiring PCI admitted to the medicine ward, intensive care unit, or intensive cardiac care unit at Eras Lucknow Medical College and Hospital, Lucknow, who approved the study (approval number: ELMC&H/RCell/EC/2020/77). In this context, stable CAD refers to patients with chronic CAD, whereas unstable CAD pertains to those experiencing ACS. Table 1 illustrates the inclusion and exclusion criteria of the present study.

**Table 1:** Inclusion and exclusion criteria CAD: coronary artery disease, STEMI: ST-elevation myocardial infarction, Trop-I: troponin-I, TMT: treadmill test, COPD: chronic obstructive pulmonary disease, EF: ejection fraction

Inclusion criteria	Exclusion criteria
Age >18 years	Patients with cardiogenic shock
Gender: male and female	Patients with resuscitated cardiac arrest
Subjects diagnosed with CAD according to the cardiac evaluation protocol are as follows: cardiology evaluation protocol for CAD: A patient experiencing chest pain (angina pectoris), with or without associated breathlessness, and exhibiting at least one abnormality on the following investigations will be considered a case of CAD based on the standard criteria (adapted from the protocol from the cardiology unit). Electrocardiogram changes: STEMI, non-STEMI, positive Trop-I, and/or positive stress test (TMT)	Patients with severe liver, kidney, and lung disease (including COPD)
Patients with at least one coronary artery with more than 70% luminal diameter stenosis and required complete revascularization	Patients with morbid obesity
	Patients with severe systolic heart failure (EF <30%), severe anemia, and valvular heart disease
	Patient with arrhythmia and flutter

The sample size was calculated based on variation in 24-hour diurnal blood pressure variation (DBPV) pre- and one-week post-PCI using the following formula:

where s1 = 1.06, representing the standard deviation (SD) of the 24-hour DBPV pre-PCI, s2 = 0.95, representing the SD of the 24-hour DBPV one-week post-PCI, and d = min(s1, s1), representing the minimum of s1 and s2. The minimum difference is considered to be clinically significant. The design effect k is set to 2.0 for adjusting multiple observation confounding [8]. Considering type I error (level of significance) α = 0.05, type II error β = 10%, power of study = 90%, and data loss of 10%, the minimum sample size required for the study is 64.

Patients diagnosed with cases of CAD underwent history-taking, physical examination, and investigation. For the PCI procedure, all interventions were performed according to the current standard guidelines. Coronary angiography was performed using the Judkins method. A 5-6 F catheter was used for selective coronary angiography through the radial artery. Two or more experienced senior physicians evaluated the coronary arterial lesions using multi-position projections. PCI and postoperative treatment were performed according to the PCI guidelines. Before and after PCI, the same antihypertensive drugs were orally administered. ABPM was monitored before PCI as well as on the first day after PCI. The procedure for ABPM was performed using a mobil-o-graph (IEM, Germany), and the results were analyzed using an HMS Client-Server 4.0 system (Informer Technologies, Inc., Los Angeles, USA). Blood pressure and heart rate were monitored for one whole day: every 30 minutes during the day (0700 to 2200 hours) and every 60 minutes during the night (2200 to 0700 hours). More than 90% of the readings were valid.

The observed parameters for ABPM included 24-hour mean systolic blood pressure (SBP), 24-hour mean diastolic blood pressure (DBP), mean day SBP (dSBP), mean day DBP (dDBP), mean night SBP (nSBP), mean night DBP (nDBP), ABP variability (BPV), and blood pressure circadian rhythm.

The data collected were subjected to analysis using SPSS Statistics version 21.0 software (IBM Corp. Released 2012. IBM SPSS Statistics for Windows, Version 21.0. Armonk, NY: IBM Corp.). Chi-square, independent samples "t"- and paired "t"-tests were used to compare the data. A p-value of less than 0.05 indicated a statistically significant difference.

## Results

**Figure 1:**
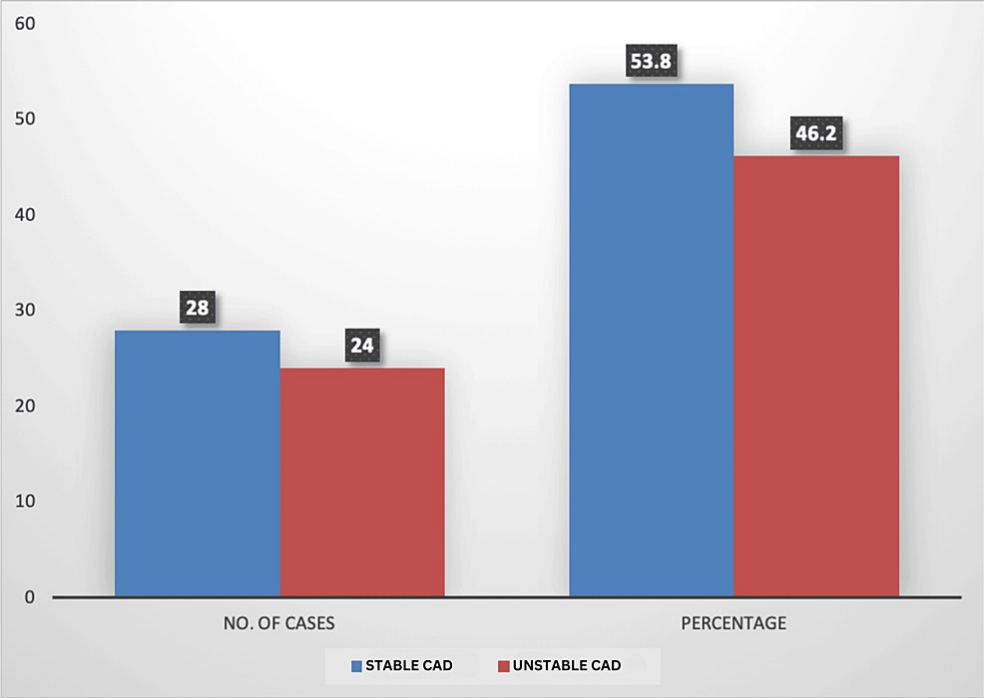
Proportional distribution of stable and unstable CAD cases CAD: coronary artery disease

The present study was carried out to study the pre- and post-PCI blood pressure variability with ABPM in patients with stable and unstable CAD. For this purpose, a total of 52 CAD patients fulfilling the inclusion criteria were enrolled in the study, and based on the nature of their disease, they were grouped into stable CAD (n=28) and unstable CAD (n=24) (Figure 1).

The demographic profile of the two study groups showed that the mean age of patients with stable and unstable CAD was 56.64±9.44 and 57.04±12.36 years, respectively. The majority of patients with stable CAD (67.9%) and unstable CAD (62.5%) were males. However, the proportion of females (37.5%) was slightly higher in unstable CAD. The baseline characteristics of patients, including basal metabolic index (BMI), blood sugar, lipid profile, cardiac troponin-I, and risk factors such as hypertension and diabetes, were recorded and found to be not statistically significant (Table 2).

**Table 2:** Demographic profile and baseline characteristics of patients in the stable and unstable CAD groups BMI: body mass index, FBS: fasting blood sugar, PPBS: postprandial blood sugar, TC: total cholesterol, TG: triglyceride, HDL: high-density lipoprotein, LDL: low-density lipoprotein, T2DM: type 2 diabetes mellitus, CAD: coronary artery disease

SN	Characteristic	Stable CAD (n=28)		Unstable CAD (n=24)		Statistical significance	
		Mean	SD	Mean	SD	t	p
1	Age	56.64	9.44	57.04	12.36	-0.132	0.896
		(range 41-75)		(range 28-79)			
2.	Sex						
	Male	19 (67.9%)		15 (62.5%)		c^2^=0.164; p=0.686	
	Female	9 (32.1%)		9 (37.5%)			
3	BMI (kg/m²)	21.50	2.61	23.24	3.95	-1.896	0.064
4	FBS (mg/dl)	124.11	53.31	109.04	26.44	1.257	0.215
5	PPBS (mg/dl)	199.39	91.84	168.17	47.27	1.502	0.139
6	HbA1c (%)	6.52	1.67	6.08	1.16	1.084	0.283
7	TC (mg/dl)	181.75	19.70	174.08	22.57	1.308	0.197
8	TG (mg/dl)	172.14	32.03	163.50	29.15	1.011	0.317
9	HDL (mg/dl)	42.29	7.50	44.67	7.09	-1.170	0.247
10.	LDL (mg/dl)	104.25	18.54	96.29	24.95	1.317	0.194
11	Troponin-I (ng/ml)	3.43	6.82	0.98	1.10	1.733	0.089
		No.	%	No.	%	c2	P
12.	Hypertension	11	39.3	10	41.7	0.030	0.862
13.	T2DM	9	32.1	6	25.0	0.321	0.571

The comparison of blood pressure variability pre- and post-PCI among stable and unstable CAD cases is shown in Table 3. Prior to and after PCI, the mean diurnal index of SBP among stable CAD patients was 14.62±6.62% and 13.78±6.17%, respectively. There was no significant difference in all the SBP measurements (p>0.05) before and after the intervention. However, the mean diurnal index was significantly lower following PCI compared to that before PCI (p=0.019).

**Table 3:** Comparison of blood pressure variability pre-and post- PCI in stable and unstable CAD cases CAD: coronary artery disease, DI: diurnal index, SBP: systolic blood pressure, DBP: diastolic blood pressure, DT: daytime, NT: nighttime

SN	Variable	Stable CAD (n=28)									Unstable CAD (n=24)					
		Pre			Post			Significance			Pre		Post		Significance	
		Mean (mmHg)	SD (mmHg)		Mean (mmHg)	SD (mmHg)		‘t’	‘p’		Mean (mmHg)	SD (mmHg)	Mean (mmHg)	SD (mmHg)	‘t’	‘p’
SBP																
1	Peak DT	139.79	17.45	137.21		13.81	1.801		0.083	143.04		16.44	138.50	10.97	2.326	0.029
	Peak NT	135.71	16.97	134.18		13.36	1.247		0.223	138.38		14.83	135.46	12.31	1.585	0.127
2.	Average DT	131.00	16.76	128.04		12.23	1.931		0.064	132.63		15.62	128.25	10.93	3.077	0.005
	Average NT	128.00	15.84	126.11		12.30	1.264		0.217	128.46		17.47	125.79	12.78	1.170	0.254
3.	Min DT	121.39	16.97	118.96		11.70	1.359		0.185	123.33		15.81	120.21	10.30	1.762	0.091
	Min NT	119.04	14.79	117.21		12.58	1.255		0.220	119.67		17.06	117.67	11.45	0.855	0.401
4.	DI	14.62	6.63	13.78		6.17	2.499		0.019	16.71		5.83	15.72	5.25	4.554	<0.001
DBP																
1	Peak DT	91.04	7.78	87.04		4.97	3.461		0.002	90.75		8.67	87.17	5.53	3.827	0.001
	Peak NT	89.21	8.88	85.36		5.59	2.527		0.018	88.71		8.14	85.00	6.67	4.207	<0.001
2.	Average DT	83.71	8.21	87.11		18.75	-0.833		0.412	83.13		8.64	82.54	17.26	0.144	0.887
	Average NT	81.89	8.57	78.68		6.27	2.530		0.018	81.00		9.34	78.88	7.14	1.340	0.193
3.	Min DT	74.43	9.40	73.39		5.75	0.918		0.367	75.38		10.18	72.08	6.16	2.194	0.039
	Min NT	74.11	7.72	71.82		6.29	1.957		0.061	73.04		9.98	71.71	6.65	0.739	0.467

On the other hand, the mean diurnal index of SBP among unstable CAD patients, prior to and after PCI, was found to be 16.71±5.83% and 15.72±5.25%, respectively. The comparison between pre- and post-PCI SBP showed a significant change in the peak daytime, average daytime, and diurnal index only (p<0.05), but other measurements were not statistically significant (p>0.05).

In DBP, a statistically significant change following PCI was observed for peak daytime, peak nighttime, and average nighttime values only (p<0.05) among stable CAD patients, whereas in unstable CAD patients, a statistically significant change was seen for peak daytime, peak nighttime, and minimum daytime values only (p<0.05).

Table 4 depicts the comparison of SBP and DBP variability between stable and unstable CAD cases before and after PCI. Pre- and post-PCI, there were no statistically significant differences between the two groups for any of the SBP and DBP parameters (p>0.05).

**Table 4:** Comparison of SBP and DBP variability between stable and unstable CAD cases before and after PCI CAD: coronary artery disease, DI: diurnal index, SBP: systolic blood pressure, DBP: diastolic blood pressure, DT: daytime, NT: nighttime, PCI: percutaneous coronary intervention

SN	Variable	Pre PCI						Post PCI					
		Stable CAD (n=28)		Unstable CAD (n=24)		Significance		Stable CAD (n=28)		Unstable CAD (n=24)		Significance	
		Mean (mmHg)	SD (mmHg)	Mean (mmHg)	SD (mmHg)	"t"	"p"	Mean (mmHg)	SD (mmHg)	Mean (mmHg)	SD (mmHg)	"t"	"p"
SBP													
1	Peak DT	139.79	17.45	143.04	16.44	-0.689	0.494	137.21	13.81	138.50	10.97	-0.367	0.715
	Peak NT	135.71	16.97	138.38	14.83	-0.597	0.553	134.18	13.36	135.46	12.31	-0.357	0.723
2	Average DT	131.00	16.76	132.63	15.62	-0.360	0.721	128.04	12.23	128.25	10.93	-0.066	0.948
	Average NT	128.00	15.84	128.46	17.47	-0.099	0.921	126.11	12.30	125.79	12.78	0.091	0.928
3	Min DT	121.39	16.97	123.33	15.81	-0.424	0.673	118.96	11.70	120.21	10.30	-0.404	0.688
	Min NT	119.04	14.79	119.67	17.06	-0.143	0.887	117.21	12.58	117.67	11.45	-0.135	0.893
4	DI	14.62	6.63	16.71	5.83	-1.200	0.236	13.78	6.17	15.72	5.25	-1.213	0.231
DBP													
1	Peak DT	91.04	7.78	90.75	8.67	3.461	0.002	87.04	4.97	87.17	5.53	3.827	0.001
	Peak NT	89.21	8.88	88.71	8.14	2.527	0.018	85.36	5.59	85.00	6.67	4.207	<0.001
2.	Average DT	83.71	8.21	83.13	8.64	-0.833	0.412	87.11	18.75	82.54	17.26	0.144	0.887
	Average NT	81.89	8.57	81.00	9.34	2.530	0.018	78.68	6.27	78.88	7.14	1.340	0.193
3	Min DT	74.43	9.40	75.38	10.18	0.918	0.367	73.39	5.75	72.08	6.16	2.194	0.039
	Min NT	74.11	7.72	73.04	9.98	1.957	0.061	71.82	6.29	71.71	6.65	0.739	0.467

Table 5 shows the pattern of dipping at pre- and post-PCI in stable and unstable CAD pectoris cases. In the stable CAD group, before PCI, most patients were dippers (67.9%), followed by extreme dippers (17.9%), and reverse or non-dippers (7.1% each). Also, according to PCI, most patients were dippers (71.4%), followed by extreme dippers (14.3%) and reverse or non-dippers (7.1% each). Statistically, there was no change in the dipping pattern according to PCI (p=0.987). In the unstable CAD group, both before and after PCI, the majority of patients (62.5%) were dippers, followed by extreme dippers (25%), non-dippers (8.3%), and reverse dippers (4.2%). The dipping pattern did not change according to PCI (p=1). The majority of patients in both groups were dippers and extreme dippers. There was no significant difference between the two groups (p=0.899).

**Table 5:** Pattern of dipping at pre- and post-PCI in stable and unstable angina pectoris cases CAD: coronary artery disease, PCI: percutaneous coronary intervention

	Parameter	Stable CAD (n=28)		Unstable CAD (n=24)		X2	p-value
		No.	%	No.	%		
Pre	Reverse dippers	2	7.1	1	4.2	0.591	0.899
	Non-dippers	2	7.1	2	8.3		
	Dippers	19	67.9	15	62.5		
	Extreme dippers	5	17.9	6	25.0		
Post	Reverse dippers	2	7.1	1	4.2	1.147	0.766
	Non-dippers	2	7.1	2	8.3		
	Dippers	20	71.4	15	62.5		
	Extreme dippers	4	14.3	6	25.0		
p-value		X2=0.137; p=0.987		X2=0; p=1.000			

## Discussion

Merely recording a blood pressure reading in the clinic to diagnose or predict a cardiovascular problem is no longer accepted. Repeated measurements are required to ensure accuracy. In recent decades, particularly since the late 1980s, the importance of circadian variability in blood pressure and heart rate [9-10] has evolved from a mere indicator of hypertension to a physiological marker of various disease patterns and health states, becoming an important research topic. In the following decades, the role of circadian variability of blood pressure and heart rate has been studied in various diseases, including renal diseases [11], headache and neuropathic pain [12], behavioral patterns [13], and, most importantly, metabolic and cardiovascular diseases [14-16]. The role of circadian variability in blood pressure in relation to CAD and its various complications has also been extensively studied [17-20], yet there are limited studies evaluating post-PCI changes and no study comparing them between stable and unstable CAD patients. Thus, the present study was performed to explore the effects of PCI on the ABPM of patients with stable and unstable CAD undergoing treatment before and after PCI.

In the present study, there were no significant differences in SBP and DBP parameters and patterns of SBP decline between the two study groups, both before and after PCI. Concerning within-group changes, patients in the stable CAD group showed no significant change in SBP patterns after PCI, except for a significant decrease in the daily index. Conversely, patients in the unstable CAD group showed a significant decrease in peak diurnal values, average diurnal values, and diurnal V-blood pressure fluctuations after PCI. In both groups, no significant change in the dip pattern was observed after PCI compared with the status before PCI. Additionally, there was no significant difference in the dip patterns of the two groups, both before and after PCI. In comparison with the present study, Cay et al. [21], who evaluated six follow-up examinations of patients who underwent PCI for CAD for the degree of restenosis, reported that the systolic and diastolic SD and the values of the coefficient of variation for the 24-hour average during the day and night (except for the diastolic VC at night) were significantly higher in the restenosis group than in the control group. In contrast to the study by Cay et al., the present study evaluated the slump pattern, but no significant change within or between groups was detected (except for a change in the diurnal index in the stable CAD group) when evaluated on the first day after PCI. Compared with Cay et al., the time to reassess change after PCI was much shorter and showed no significant difference within or between groups. In another study, a comparison between CAD and non-CAD women showed significant differences in nocturnal SBP, SBP drop, and DBP drop. However, in the present study, the two CAD groups were distinguished according to stable and unstable values [21].

Regarding the differences between stable and unstable CAD groups, there are few studies reporting a difference in BPV patterns. Harefa et al., who investigated the influence of 24-hour BPV on serious cardiac events (MACE) in patients with acute MI, reported no significant difference in the BPV pattern of patients with stable or unstable CAD. In addition, they also found no effect of the type of MI patients on MACE [22].

The results of the present study are consistent with the observations of Yang et al., who examined the changes in ambulatory blood pressure (ABP) one to three days before PCI, three to six days after PCI, and one month after PCI and found that the pattern immediately after PCI ABP (three to six days after PCI) was not significantly different from the pattern before PCI ABP, and it took one month for this pattern to change into a statistically significant change [8]. One of the limitations of the present study was that the evaluation was performed only one day after PCI, and we were not able to perform follow-ups at longer intervals. This limitation was due to the COVID-19 pandemic, during which hospital services were impaired for a prolonged period, and even after the resumption of outpatient department services, most patients were discouraged by CAD from participating in such an evaluation, which was not an essential part of their follow-up because of their higher vulnerability to pandemic transmission and adverse outcomes. Her et al. [23], in a study that limited the evaluation of ABPM for ward-to-cath lab blood pressure before PCI, reported that the proportion of patients with large differences in SBP (>20 mmHg) was 38.5%. In the present study, we did not evaluate the changes before PCI for two reference periods but instead focused on the changes after PCI in ABP to assess the immediate effects of PCI and found that the immediate changes after PCI in the pattern of ABP generally did not reflect the effects of PCI. The likely reason for this may be the time required for the autonomic nervous system to adjust to the newly altered status of CAD. In addition, the rule of prophylaxis treatment during the period immediately after PCI may also be considered a confounding factor.

In the present study, we also focused on the association of BPV with in-hospital mortality. Fortunately, there was no mortality, so we cannot comment on this. Considering the low in-hospital mortality rate in patients undergoing PCI, this is not the case. Large-scale epidemiologic studies report in-hospital mortality ranging from 1.1% to 3.3%, with a median of 1.9% [24]. In a small study that primarily included patients not at high risk, the likelihood of in-hospital mortality was relatively low, and incidentally, in the present study, mortality did not occur in either study group. An association between ABP and mortality was found in studies with a long follow-up period [25] and more frequently in patients with ACS [26]. In the present study, patients with cardiogenic shock, resuscitated cardiac arrest, severe liver, kidney, and lung disease (including chronic obstructive pulmonary disease), morbid obesity, severe systolic heart failure (ejection fraction <30%), severe anemia, valvular heart disease, arrhythmias, and heart flutter were excluded, so they had a safer profile. In contrast to the results of the present study, Shah et al. [27] found a significant reduction in mean blood pressure (SBP and DBP) and percentage changes in blood pressure after PCI. However, their study was a follow-up study, and the results they reported did not reflect the evaluation one day after PCI as in the present study. In a previous study, Denardo et al. showed that revascularization produced most blood pressure changes within six weeks after the procedure. However, unlike the present study, in which the final outcome was observed 24 hours after PCI, they had a much longer mean follow-up time (32.9 months) [28].

In the present study, both stable and unstable CAD showed a predominance of dippers in the assessments before and after PCI. In comparison, Kenchi et al. found that only 41% of their patients were dippers and found no significant difference between dippers and non-dippers in age, ejection fraction, presence of DM, severity of CAD, type of presentation, and pulse wave velocity [29]. A similar nonsignificant difference was observed for the CAD type in the present study, existing both before PCI and after PCI. Like the present study, Nathani found a predominance of dippers (62.8%) in her study of 43 STEMI patients, comparable to the high prevalence of dippers in both the unstable and stable CAD groups in the present study [30].

Within their limitations, the results of the present study are comparable with most previous studies. However, further long-term studies with a larger sample could help investigate some of the issues that could not be adequately assessed due to the pandemic effect.

## Conclusions

Overall, 46.2% of patients had unstable CAD, whereas 53.8% had stable CAD. In terms of daily peak, mean time of day, and daily index, mean SBP changed significantly before and after the study in stable CAD PCI, but significantly before and after the study in unstable CAD PCI. In stable CAD, mean DBP changed significantly before and after the PCI study at peak daytime, peak nighttime, and average nighttime times, whereas in unstable CAD, mean DBP changed significantly before and after the PCI study at peak daytime, peak nighttime, and minimum daytime times.

In patients before and after PCI, there was no discernible difference between stable and unstable CAD in dipping patterns such as dipper, non-dipper, extreme dipper, or reverse dipper. In the 48 hours after PCI, ambulatory blood pressure indicators did not change significantly from those before PCI, as shown by a comparison of ABPM before and after PCI.
